# Fatal course of undetected Ewing-like sarcoma in a 9-year-old boy with uncharacteristic clinical presentation

**DOI:** 10.1007/s12024-021-00437-2

**Published:** 2021-11-15

**Authors:** Walther Gotsmy, Bettina Neumayer, Theo Kraus, Barbara Zellinger, Daniel Neureiter, Fabio Monticelli, Harald Meyer, Peter Hofer

**Affiliations:** 1 Department of Forensic Medicine, Paris-Lodron University of Salzburg, Salzburg, Austria; 2 Institute of Pathology, Paracelsus Medical University Salzburg, Salzburg, Austria

**Keywords:** Ewing-like sarcoma, Small blue round cell tumors, Autopsy, Diagnosis, Unexpected death

## Abstract

A 9-year-old boy collapsed shortly after complaining of shortness of breath. Despite immediate resuscitation measures, the boy died. A few weeks earlier, he had received antibiotic treatment for respiratory infection. However, the post-mortem examination revealed an advanced tumor mass of the mediastinum with infiltration of vital structures, which was identified as a small blue round neoplasm with aspects of an extramedullary Ewing-like sarcoma by supplementary histological and immunohistochemical examinations.

This dramatic clinical course of events shows that the possible presence of serious diseases should always be considered behind harmless symptoms, even in children.

## Case report

A 9-year-old boy suddenly complained of shortness of breath late in the evening, ran out of his room and collapsed a little later in the entrance area of the house. The child had been suffering from respiratory infection for about two weeks. To this end, the general practitioner (GP) had started antibiotic therapy with Cefaclor and Phenoxymethylpenicillin. Immediate resuscitation measures by the parents and the subsequently arriving paramedics were unsuccessful, and the emergency doctor could only determine the boy’s death. Due to the unclear cause of death and the possibility of contagious diseases, the public prosecutor ordered a forensic autopsy, which was performed two days after the lethal incident.

## Autopsy results

An autopsy of the boy, who had a body length of 144 cm and a body weight of 37 kg, was performed. Overall, the body was in a proper state of development and good nutritional condition.

**Fig. 1:**
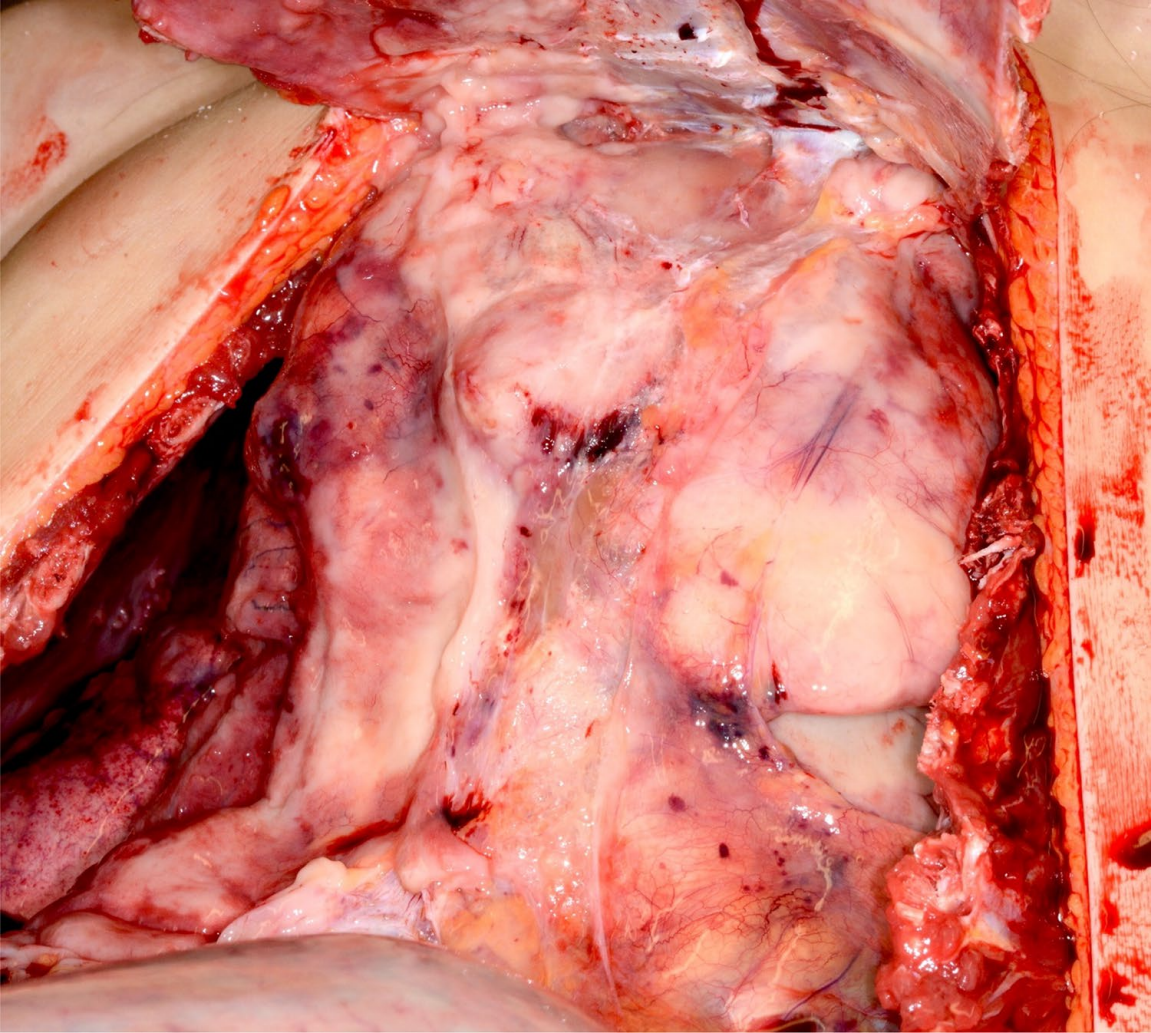
Overview of the mediastinum after opening the thorax. Note the massively retracted lungs

**Fig. 2:**
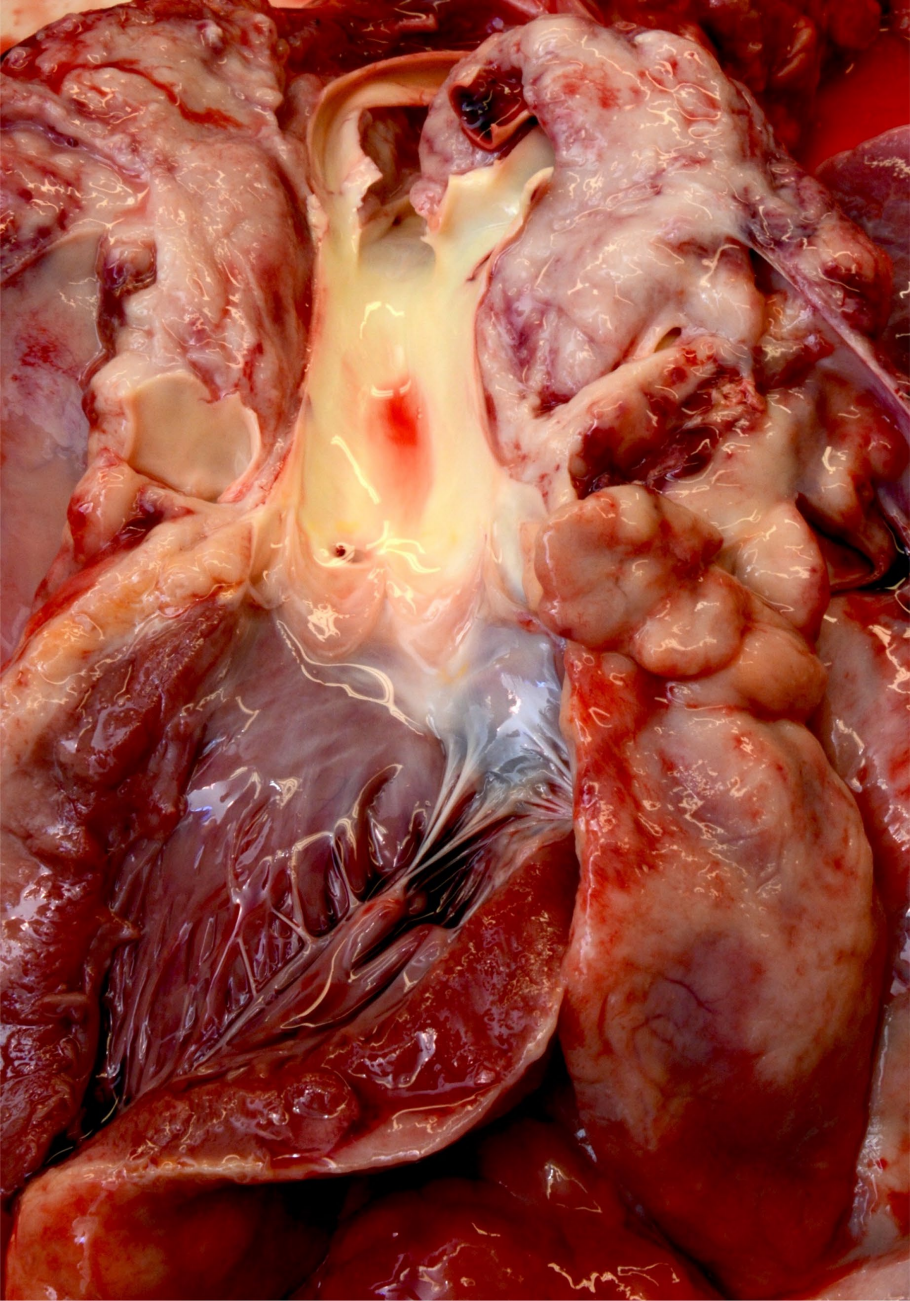
Sheathing of the aorta and its branching vessels by tumor masses

After opening the thoracic cavity, a large tumor mass occupying almost the entire mediastinal space was found. It surrounded all organs of the mediastinum and was palpable up to the posterior part of the thoracic cavity (Fig. 1). In the posterior thoracic cavities, especially on the right side, infiltrations of presumably neoplastic tissue were seen in the course of the intercostal spaces. On the right side of the anterior chest wall, there was yellow-brownish, creamy softened tissue. Regular muscle tissue was no longer delineated here. In this respect, it could be assumed that the tumor had originated on the right thoracic wall. The tumor encased the trachea, the esophagus, the aorta and the carotid arteries, with subsequent compression of these structures (Fig. 2).

**Fig. 3:**
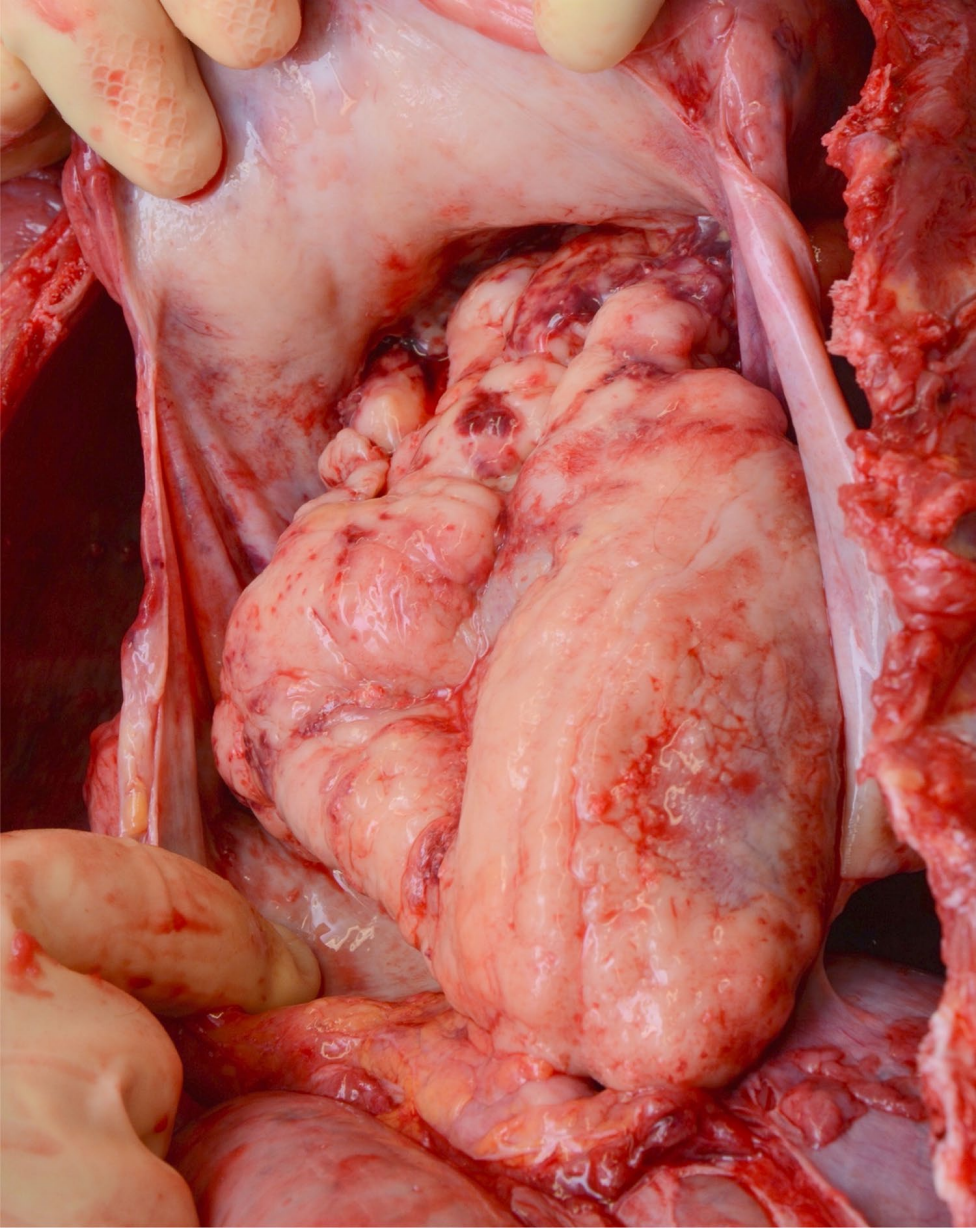
Heart almost completely encased in tumor mass after opening of the pericardium

**Fig. 4:**
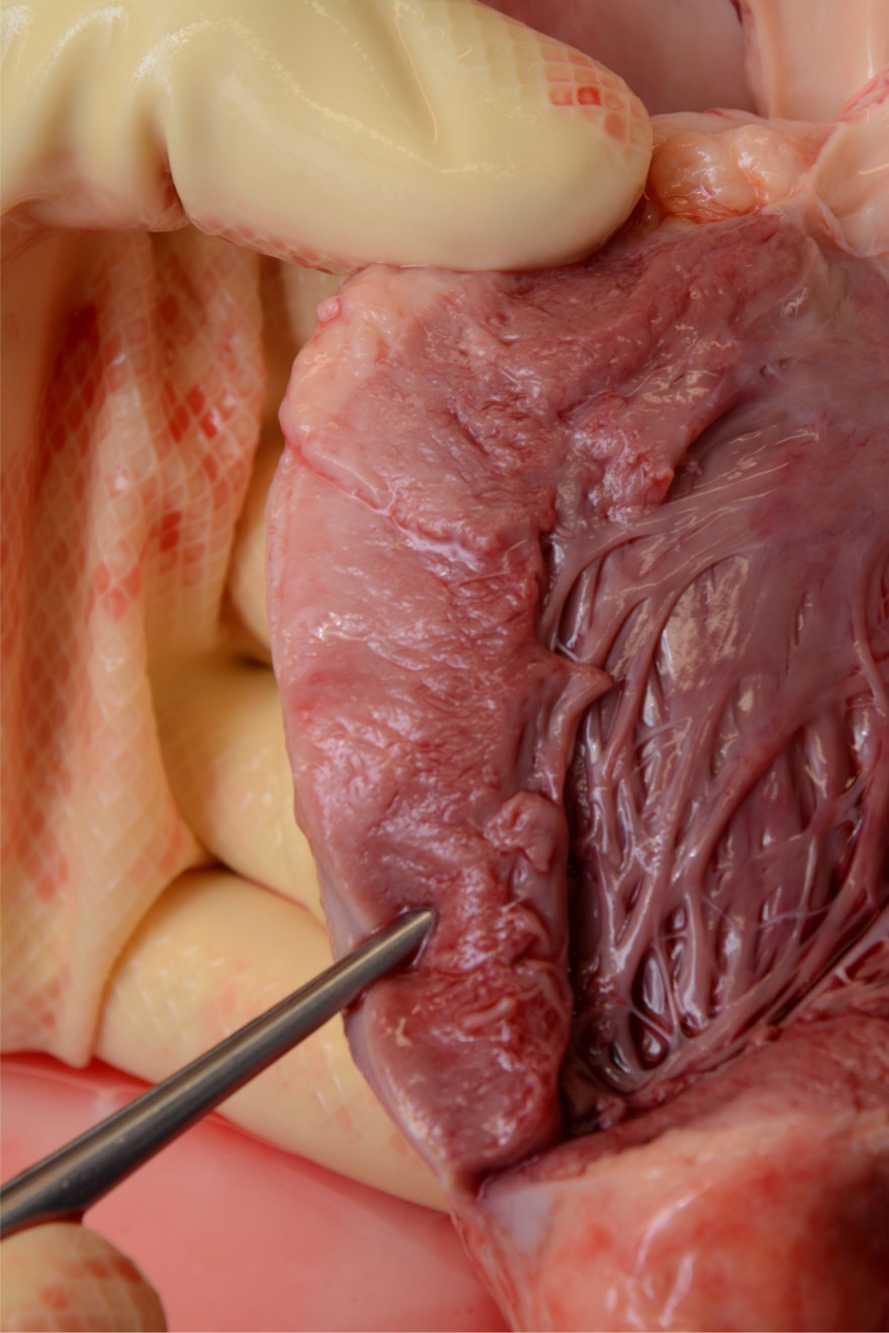
Tumor ingrowth into myocardium

The heart weighed 320 g, which is almost three times higher than healthy hearts of children in the same age group [1]. This was caused by massive infiltration of tumor tissue. The epicardium showed clearly accentuated vessels. In addition, the heart was almost completely covered by a white, rough tumor mass. On sections through the myocardium, yellow–brown infiltrations were visible, especially in the area of the left ventricle (Figs. 3 and 4).

In addition, there was a purulent infection of the respiratory tract as well as chest effusions. Bilateral retracted lungs were present and the right lung in particular displayed fleshy white infiltrates into the tissue.

No other significant illnesses or signs of trauma were found during the autopsy. Thus, according to the autopsy results, the boy died of cardiorespiratory failure due to malignant tumor disease in an advanced stage with extensive heart involvement.

## Histological and immunohistochemical findings

**Fig. 5:**
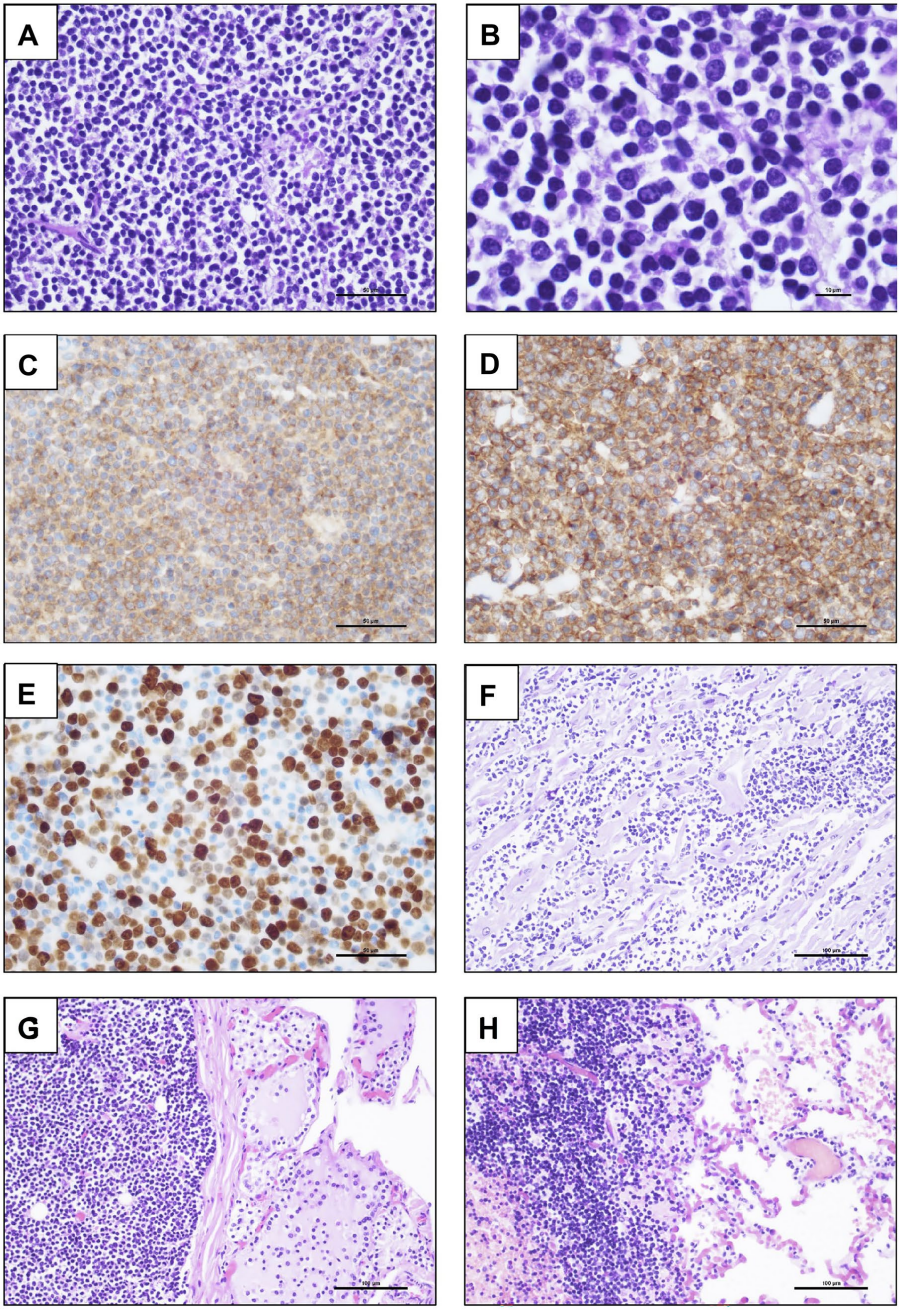
Histomorphological features **A-B**: HE and immunohistochemical expression pattern (C: CD10, D: CD99, E: Ki67) of the small-blue-round-cell neoplasm **A-E** of the large mediastinal mass with infiltration of heart **F**: HE, thyroid gland **G**: HE and the lung **H**: HE. Magnification: 200 × **F** to **H**, 400 × **A**, **C**, **D** and **E** and 1000 × **B**

Histomorphological investigation of the mediastinal tumor revealed a neoplasm showing a solid growth pattern and consisting of small round tumor cells with hyperchromatic and prominent nuclei and narrow cytoplasm (see Fig. 5A and B). The consecutive histomorphological analysis of the organs revealed a diffuse infiltration of the heart (F), thyroid gland (G) and the lung (H) by this small blue round neoplasm as shown in Fig. 5.

The malignant tumor was additionally characterized by intensive immunohistochemical staining. The antibodies AE1/AE3, AFP, ALK, CD1a, CD3, CD10, CD20, CD30, CD34, CD43, CD45 LCA, CD68, CD79a, CD99, CD117, Chromogranin-A, beta-HCG, INI-1, Ki67, MAP2, Myogenin, MPOX, PLAP, Podoplanin, S100, Synaptophysin, TdT and WT1 were applied to already established pathological routines on the two immunohistochemical staining platforms Benchmark Ultra (Roche®, Vienna, Austria) and Dako OMNIS (Agilent Technologies®, Vienna, Austria). The tumor cells showed a strong immunohistochemical positivity for CD10, CD99 and INI-1, with all other applied antibodies remaining negative. The Ki67-associated proliferation activity of the small blue tumor cells was up to 80% in a hot spot area (as demonstrated in Fig. 5C-E). The fluorescence in-situ-hybridization with a EWSR1 dual color break-apart probe (ZytoVision®, Bremerhaven, Germany) was performed whereby no EWSR1 gene re-arrangement could be detected in the analyzed tumor specimen.

## Molecular findings

Thus, further molecular pathological investigations were performed. RNA and DNA was isolated under histomorphological control.

Mutation analysis was performed by next generation sequencing using the AmpliSeq Myeloid® Panel (Illumina®). DNA segments of the hot-spot regions of tumor-relevant genes were specifically enriched and sequenced on the MiniSeq platform (Illumina®) (2 × 151 bp paired end). Bioinformatic data analysis was performed using SeqNext module of JSI SeqPilot ™ (version 4.4.0 build 502), comparing the obtained gene sequences with the human reference sequence GRCh37 (hg19). Variants of unclear significance (VUS) and certainly disease-relevant mutations (class 4 and 5) are reported. The minimum coverage for the analysis was set at 500 x, variants below 5% allele frequency were not considered. Nucleotide positions were numbered according to HGVS recommendations [2].

Fusion analysis was performed by next generation sequencing using the Archer® Fusion Plex® Expanded Sarcoma Panel (Archer®). RNA segments were targeted enriched and sequenced on the MiniSeq platform (Illumina®) (2 × 151 bp paired end). Bioinformatic data analysis was performed using ArcherDX Analysis 6.2 site.

The relevant molecular pathological findings were:

DNA: All genes present in the myeloid NGS panel were analyzed, whereby there was no evidence of unclear and/or clinically relevant variants (class 3 to 5).

RNA: Both RNA samples were of low quality and the resulting cDNA was poorly amplifiable. No EWSR1 fusion was detected. Forty-one different fusion products of unknown biological relevance were additionally found, all with low coverage (5 to 18 reads). All detected fusions (as shown in the supplementary file) were additionally checked with the Fusion Gene annotation DataBase (https://ccsm.uth.edu/FusionGDB/index.html, last access at 27.08.2021) [3], whereby none of these could be found in the published database.

In summary, the deep molecular analysis revealed no specific mutations or fusions of the investigated DNA and RNA, especially the EWSR1 fusion. In detail, the myeloid panel gives no evidence for a specific drive mutation for a myeloid neoplasm on DNA level. The definitive role of the other RNA-fusion remains unclear. In recent years, a group of Ewing-like sarcomas have been described which share morphological, immunohistochemical and clinical similarity with the classical Ewing-sarcoma, whereby the typical translocation between a gene of the RNA‐binding TET family (EWSR1 or FUS) with a gene of the ETS‐transcription family is missing by means. Interestingly, molecular methods could identify subgroups by specific molecular driving events like CIC or BCOR-rearranged sarcoma [4–6]. Unfortunately, we were not able to find one of these novel fusions in our case, which may have possibly been influenced by post-mortem degradation, especially of the RNA. Thus, integrating histomorphology, immunophenotype and molecular analysis alternative explanations [7] were excluded as far as possible and a working diagnosis of Ewing-like sarcoma was finally made.

## Discussion

Ewing sarcomas (ES) and Ewing-like sarcomas represent a clinically and immunohistochemical heterogeneous group, but are uniform at the molecular level and are classified as small blue round cell tumors, which preferentially occur in childhood and adolescence [8]. Although Ewing-like sarcoma has some differences from ES, it is often still considered a subtype because of the great similarities to it. However, there is growing evidence that Ewing-like sarcoma should be considered as its own type of sarcoma and not just a subtype of ES.

In Austria, an average of 4.7 children and adolescents per 1,000,000 per year were diagnosed with ES between 2009 and 2018 [9]. It should be noted that there is no differentiation from Ewing-like sarcomas, which is why these are counted as ES in the statistics.

After osteosarcoma, ES is the second most common malignant bone tumor in children. In 80% of cases, patients are 20 years old or younger, with a peak between 10 and 15. Boys are affected more often; there is also a strong predilection for Caucasians, while those of African descent are affected significantly less often [10]. The primary tumors occur most frequently in the long tubular bones and the pelvic bones [11].

In adulthood, tumors mostly occur extramedullary in the deep paravertebral soft tissue and the proximal sections of the extremities [12], whereby manifestation in the chest wall in children or young adults [13] and of the mediastinum [14] is recently described in the literature.

Clinically, the first symptoms are usually pain, which is often misinterpreted as so-called growing pains, which often leads to the fact that in up to a quarter of the cases at the first diagnosis of ES, the tumor has already metastasized, mainly to the lungs and the bones or bone marrow [8].

Patients often describe local symptoms such as pain, swelling, hyperemia or induration. In later stages, pathological fractures may occur as a result of bone metastases. In cases of extramedullary ES originating in the chest wall, pleural infiltration with pleurisy may be observed. In addition, anemia and leukocytosis are common, as is as an increase in lactate dehydrogenase and C-reactive protein.

Bone destruction can often be detected on plain radiographs; in the case of soft tissue infiltration, computed tomography often proves helpful [15]. Histologically, typical small round cells with a blurred, faintly stained cytoplasm are found. In between, there is partly edematous loosened connective tissue [16].

Therapy consists of neoadjuvant and adjuvant chemotherapy as well as surgical removal of the affected tissue and radiotherapy and, if necessary, high-dose chemotherapy [17].

With multimodal therapy, cure rates of > 70% can generally be achieved for localized diseases, although overall survival decreases dramatically depending on tumor location, metastasis, and volume [18, 19].

Although there has been a marked improvement in the results of treatment in recent decades, the long time between the first symptoms and the correct diagnosis with subsequent therapy remains a major clinical problem [20].

In the present case, the post-mortem examination findings were unexpected, based on the available plain medical history and the unremarkable external examination of the body. However, because of the advanced tumor disease, the symptoms described before death were plausible due to the location and extent of the chest tumor tissue. Due to the inconspicuous medical record and the boy’s unremarkable behavior until about 2–3 weeks before his death, no preliminary imaging examinations or laboratory blood tests were performed that could have revealed the dramatic findings.

The public prosecutor’s office was concerned with clarifying the question of whether the GP who had treated the boy for a respiratory infection should have positively diagnosed the serious illness, and whether the death of the child could have been prevented. Due to the advanced stage of the disease, even with the appropriate imaging, the prognosis would have been considered inauspicious. In this respect, there was no cause for reproaching the GP for medical malpractice.

Although ES-group is a very rare disease overall, it is the second most common bone tumor in children and adolescents. In this respect, the possibility of malignancy should be considered in children, and appropriate examinations should be arranged. In particular, the possibility of blood tests should be considered and, after weighing the appropriate risk, the possibility of consecutive imaging procedures.

In addition, in the case of the unexpected death of children and adolescents, a clinical and/or forensic autopsy should always be sought as a number of cases are also reported with unexpected death due to undiagnosed malignancy of the brain, heart, liver and adrenal gland [21–27]. Here, we present the first case of an undetected, local, very advanced extramedullary Ewing-like sarcoma of the mediastinum. The clarification of the cause of death naturally has an effect on the cause of death statistics. On the other hand, it is often important for relatives to clarify ambiguities and to be able to close the mourning process.

## Key points

1. The present case shows that even advanced sarcomas can be practically asymptomatic.
2. Ewing-like sarcomas often present as histomorphologically similar to Ewing sarcoma, but appear to represent a distinct form of sarcoma.
3. A correct diagnosis always requires further immunohistochemical and molecular pathological examinations in addition to the classical histolopathology.
4. Even though Ewing(-like) sarcoma is a rare disease, the possibility of such a disease should always be considered, even if the clinical picture is unusual.
5. In the present case, it was not possible for the physician to make the correct diagnosis because of the unusual findings. However, the forensic physician should always consider the possibility of medical malpractice.
